# Advances and Challenges in Understanding MicroRNA Function in Tauopathies: A Case Study of miR-132/212

**DOI:** 10.3389/fneur.2020.578720

**Published:** 2020-09-29

**Authors:** Emmanuelle Boscher, Julia Hernandez-Rapp, Serena Petry, Remi Keraudren, Sara Rainone, Andréanne Loiselle, Claudia Goupil, Andréanne Turgeon, Isabelle St-Amour, Emmanuel Planel, Sébastien S. Hébert

**Affiliations:** ^1^Axe Neurosciences, Centre de Recherche du CHU de Québec-Université Laval, Québec, QC, Canada; ^2^Département de Psychiatrie et Neurosciences, Université Laval, Québec, QC, Canada

**Keywords:** tau, microRNA, PS19, miR-132, tauopathies

## Abstract

In the past decade, several groups have reported that microRNAs (miRNAs) can participate in the regulation of tau protein at different levels, including its expression, alternative splicing, phosphorylation, and aggregation. These observations are significant, since the abnormal regulation and deposition of tau is associated with nearly 30 neurodegenerative disorders. Interestingly, miRNA profiles go awry in tauopathies such as Alzheimer's disease, progressive supranuclear palsy, and frontotemporal dementia. Understanding the role and impact of miRNAs on tau biology could therefore provide important insights into disease risk, diagnostics, and perhaps therapeutics. In this Perspective article, we discuss recent advances in miRNA research related to tau. While proof-of-principle studies hold promise, physiological validation remains limited. To help fill this gap, we describe herein a pure tauopathy mouse model deficient for the miR-132/212 cluster. This miRNA family is strongly downregulated in human tauopathies and shown to regulate tau *in vitro* and *in vivo*. No significant differences in survival, motor deficits or body weight were observed in PS19 mice lacking miR-132/212. Age-specific effects were seen on tau expression and phosphorylation but not aggregation. Moreover, various miR-132/212 targets previously implicated in tau modulation were unaffected (GSK-3β, Foxo3a, Mapk1, p300) or, unexpectedly, reduced (Mapk3, Foxo1, p300, Calpain 2) in miR-132/212-deficient PS19 mice. These observations highlight the challenges of miRNA research in living models, and current limitations of transgenic tau mouse models lacking functional miRNA binding sites. Based on these findings, we finally recommend different strategies to better understand the role of miRNAs in tau physiology and pathology.

## Introduction

Tauopathies comprise a group of ~30 neurodegenerative disorders characterized by the pathological accumulation of hyperphosphorylated and insoluble tau in neurons and/or glia (1). In humans, the *MAPT* gene encoding tau contains 16 exons, with the first exon as part of the promoter region and last exons comprising the 3′ untranslated region (3′UTR). The tau mRNA transcript undergoes different steps of regulation including fine-tuning of expression, alternative splicing of exons 2, 3, and 10, and multiple levels of phosphorylation (1). These modifications play a central role in tau function related to the binding and stabilization of microtubules (2). While rare mutations in the *MAPT* gene underlie familial forms of disease (e.g., frontotemporal dementia with parkinsonism-17), the majority of tauopathies are sporadic and of unknown origin. The most prevalent tauopathy is Alzheimer's disease (AD), where tau aggregates into neurofibrillary tangles (NFTs) in conjunction with amyloid-β (Aβ) plaques (3). So far, it remains uncertain which mechanisms surrounding tau biology contribute to brain degeneration and clinical outcomes.

The small (~21 nts) non-coding microRNAs (miRNAs) play a fundamental role in brain development, function, and survival (4, 5). They function as part of the endogenous RNA-induced silencing complex (RISC) to control protein output. This occurs by binding to mRNA transcripts within the 3′UTR to promote translational repression or mRNA degradation. Interestingly, the brain contains a rich repertoire of miRNAs, some of which go awry in tauopathies. While affected miRNAs have been associated with tau pathology in humans and animal models, the cause-consequence relationship between these factors remains ill defined. Nonetheless, specific miRNAs have emerged as promising diagnostic and therapeutic targets in tauopathies (6–8).

Several methods currently exist to study miRNA:mRNA interaction and biological function (9). These range from bioinformatic predictions, 3′UTR reporter assays, overexpression and inhibition studies, to cross-linking with immunoprecipitation (CLIP). The most common and straightforward approach is introducing a mutation within the miRNA target site (in particular the seed sequence) to inhibit miRNA:mRNA binding and gene expression regulation. This strategy is however quite challenging *in vivo* with only one known report in the mammalian brain (10), unrelated to tau. Most miRNA literature is therefore based on indirect or artificial paradigms that await physiological validation.

In this Perspective article, we provide an overview of advances related to the regulation of tau by miRNAs. As the reader will notice, the literature is promising but lacks consistency and *in vivo* validation. To aid in this effort, we also describe herein PS19 mice deficient for the miR-132/212 cluster. This is the first description of a pure tauopathy mouse model genetically deficient for specific miRNAs. Some paradoxical results obtained in this model prompted us to address certain “barriers” regarding experimental reproducibility and propose guidelines to help move forward this line of research.

## Perspective Article

### Evidence That tau Is a MicroRNA Target

The human *MAPT* gene produces two 3′UTR isoforms of 256 and 4,163 nucleotides in length (11). The longest isoform is conserved and highly expressed in the brain (frontal cortex) (12). Previous studies have shown that different domains within the tau 3′UTR are important for mRNA structure, stability, and transport (13–15). Since tau is a dose-sensitive gene candidate (16, 17), and that its mis-regulation is associated with disease (18), it seems logical that different regulatory mechanisms have evolved to keep tau expression levels in check.

A simple search of common miRNA target site prediction programs (e.g., targetscan.org) reveals several conserved binding sites within the tau 3′UTR. Consistent with this, a handful of miRNAs have been shown to bind to tau mRNA, including miR-132 (19, 20), miR-34 (11), miR-186 (21), miR-219 (22), miR-362 (23), and miR-766 (23). Most groups have relied on 3′UTR luciferase reporter assays and mutagenesis to confirm gene expression regulation *in vitro*. One report could not confirm the interaction between miR-132 and its corresponding seed region however (11). Whether this is due to technical issues (type of mutagenesis, miRNA titration) or unknown regulatory mechanisms remains to be determined. Taken together, these observations provide strong evidence that tau is a *bone fide* miRNA target that now awaits *in vivo* validation using gene editing technologies. Interestingly, the ratio between tau 3′UTR isoforms seems to differ between healthy and AD brain (12, 24). Whether this results in altered miRNA regulation requires further investigation.

### MicroRNA Regulation of tau Pre- and Post-translational Modifications: Unlimited Possibilities?

As stated above, alternative splicing and phosphorylation are key elements of tau regulation and function. Nearly 15 miRNAs have been implicated so far in the *indirect* modulation of tau (8, 25). These “tau modifier” genes are mostly kinases, and include Gsk-3β [miR-132 (26), miR-125b (27), miR-124 (28), miR-219 (29, 30), miR-138 (31)], Cdk5 [miR-125b (32, 33), miR-26b (34), miR-195 (35)], Erk [miR-125b (32)], Itpkb [miR-132 (36)], Fyn [miR-369 (37), miR-106b (38)], and Rock1 [miR-146a (39)]. Tau phosphatases include: Ppp1ca [miR-125b (32)] and Ptpn1 [miR-124 (40)]. Other genes *a priori* unrelated to kinases or phosphatases include p300 [miR-132 (26)], RbFox1 [miR-132 (26)], Nos1 [miR-132 (41)], BNDF [miR-322 (42)], RARα [miR-138 (31)], Cacna1c [miR-137 (43)], Uchl1 [miR-922 (44)], and HspB8 [miR-425 (45)].

The number of miRNA targets involved in tau splicing (exon 10) is more limited. These include Ptbp1 [miR-132 (19)] and yet unidentified genes [miR-124, miR-9, miR-153, miR-137 (19)]. Overall, while some miRNAs (miR-132, miR-125b, miR-124) and target genes (GSK-3β, Cdk5) seem recurrent, no clear pathway or trigger stands out and *in vivo* validation is again largely lacking. To study miRNAs in living organisms, especially in mammals, is particularly challenging since most tau modifier genes contain several miRNA-binding sites themselves (e.g., GSK-3β 3′UTR contains ~100 putative miRNA sites). Thus, the amount of potential miRNA:mRNA networks surrounding tau biology seem almost limitless, at least in appearance.

Indeed, bioinformatics and *in vitro* paradigms need to be tested and experimentally validated using *in vivo* models recapitulating the cellular and miRNA-target interaction networks occurring in human physiology and pathology. Furthermore, not all miRNAs modulated in cultured cells are biologically relevant since expressed at low or insufficient levels *in vivo* (as with their target counterparts). Also, not all target genes are sensitive to small changes in mRNA transcript or protein levels (46–48). This said, it is likely that only a limited number of miRNAs and dosage-sensitive genes are involved in the physiological regulation of tau (see also Barrier 2 below). This adds to other potential modes of miRNA action implicating competitive endogenous RNAs (ceRNA) (49) as well as cooperative binding and target site competition (50). An important step will be to identify the functional miRNA:mRNA pairs within the biological networks in brain cells.

### A Mouse Model to Study miRNA Deficiency in Pure Tauopathies

Recent RNA deep-sequencing efforts have shown that ~50–100 miRNAs are expressed at moderate to high levels in the mouse and human brain (51–53). Several of these are enriched in neurons, glia, or other cell types (54). Given the diversity of tau pathologies, selecting a candidate miRNA, and biological model for functional studies is a daunting task. Interestingly, accumulating studies highlight the potential biological importance of specific miRNAs. For example, loss of the miR-132/212 cluster shows a strong correlation with memory decline, NFTs, and Braak (tau pathological) stages in AD (20, 26, 51, 55–59). This cluster is also downregulated in other tauopathies such as frontotemporal dementia (60) and progressive supranuclear palsy (19). Deletion of the miR-132/212 cluster, or inhibition of neuron-specific miR-132, causes an increase in tau phosphorylation or aggregation in 3xTg-AD and APP/PS1 mice (36, 41, 61). Inversely, the brain delivery of miR-132 using viruses reduced tau pathologies in 3xTg-AD (61) and PS19 (26) mice. While promising, different target genes and underlying mechanisms have been proposed, and therefore the mode of action remains unresolved.

To further build on these findings, we investigated the effects of genetically removing the miR-132/212 cluster in PS19 mice, a model of pure tauopathy that overexpresses human tau with a mutation (P301S) that causes FTD in humans (62). This model develops motor deficits, tau deposition, and neuronal loss between 6 and 12 months, with a high mortality rate at late-stage disease.

**Figure 1:**
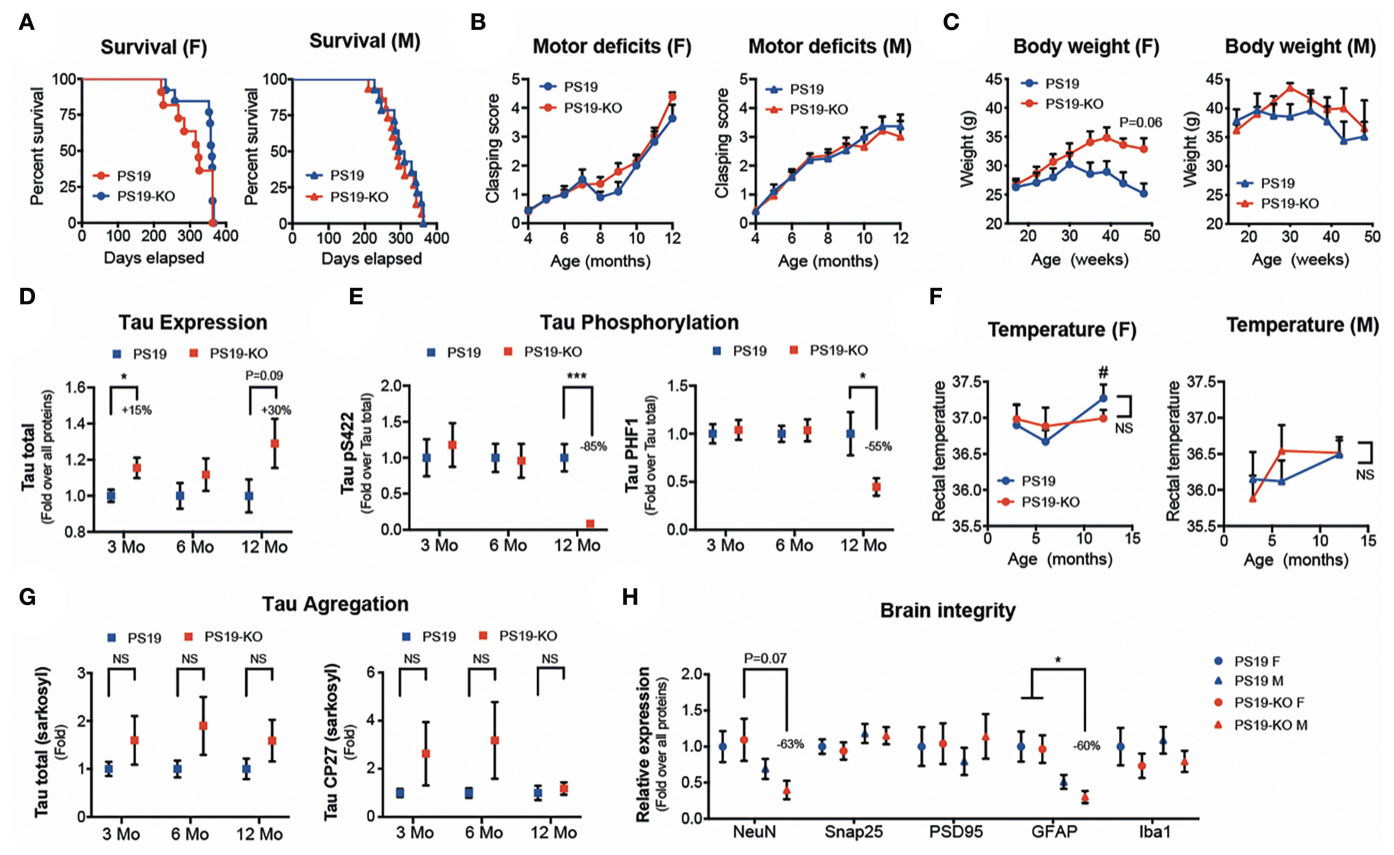
Characterization of PS19 mice lacking the miR-132/212 cluster. **(A)** Kaplan-Meier survival curves, **(B)** clasping scores, and **(C)** body weight of PS19 and PS19-KO mice that are deficient for the miR-132/212 cluster. PS19 mice (JAX No. 008169) were bred with full miR-132/212 KO mice as before (61). Graphs were divided by sex. No significant changes were observed between mouse models. Kaplan-Meier and one-way ANOVA. *N* = 6–16 mice per group. **(D)** Western blot quantifications of cortical total tau expression and phosphorylation (Ser422 and PHF1 epitopes) **(E)** at different ages (3–12 months). *N* = 12–16 mice per group, mixed sex. Unpaired *t*-test, where *P < 0.05, ***P < 0.001. **(F)** Rectal temperature at sacrifice. # denotes significant changes (multiple *t*-tests, *P* < 0.05) between PS19 males and females at 12 months. **(G)** Western blot quantifications of cortical sarkosyl-insoluble tau (Tau total and CP27) at different ages. *N* = 9–16 mice per group, mixed sex. Unpaired *t*-test. **(H)** Western blot quantifications of cortical endogenous NeuN, Snap25, PSD95, GFAP, and Iba1 in 12-month-old PS19 and PS19-KO mice. *N* = 9–10 mice per group, divided by sex. One-way ANOVA with multiple comparison, where *P < 0.05. Error bars represent SEM. In **(D-G)**, the groups of mice were analyzed separately per age.

We observed no significant differences in mouse survival (Figure 1A) and motor deficits (Figure 1B) following miR-132/212 deletion in PS19 mice (PS19 vs. PS19-KO). A trend for increased body weight was seen in 11–12-month-old PS19-KO females but not males (Figure 1C). A small but significant increase in tau expression was seen in pre-symptomatic (3 months) PS19-KO mice with a trend in aged (12 months) mice (Figure 1D). In contrast, tau phosphorylation at Ser422 and PHF1 (Ser396/Ser404) epitopes was reduced in aged PS19-KO mice (Figure 1E). This was not attributed to changes in body temperature known to influence tau phosphorylation (63) (Figure 1F). Non-significant trends were noticed in tau aggregation (sarkosyl-insoluble tau) in PS19-KO mice, owing to ~20% of KO mice with more aggregates (Figure 1G and not shown). In addition, no or minimal effects were seen on different markers of brain integrity, including NeuN (neuron), Snap25 (presynaptic), PSD95 (postsynaptic), GFAP (astrocyte), and Iba1 (microglia) (Figure 1H). Taken together, the deletion of the miR-132/212 cluster had no major effects on disease phenotypes tested with divergent effects on tau biology.

**Figure 2:**
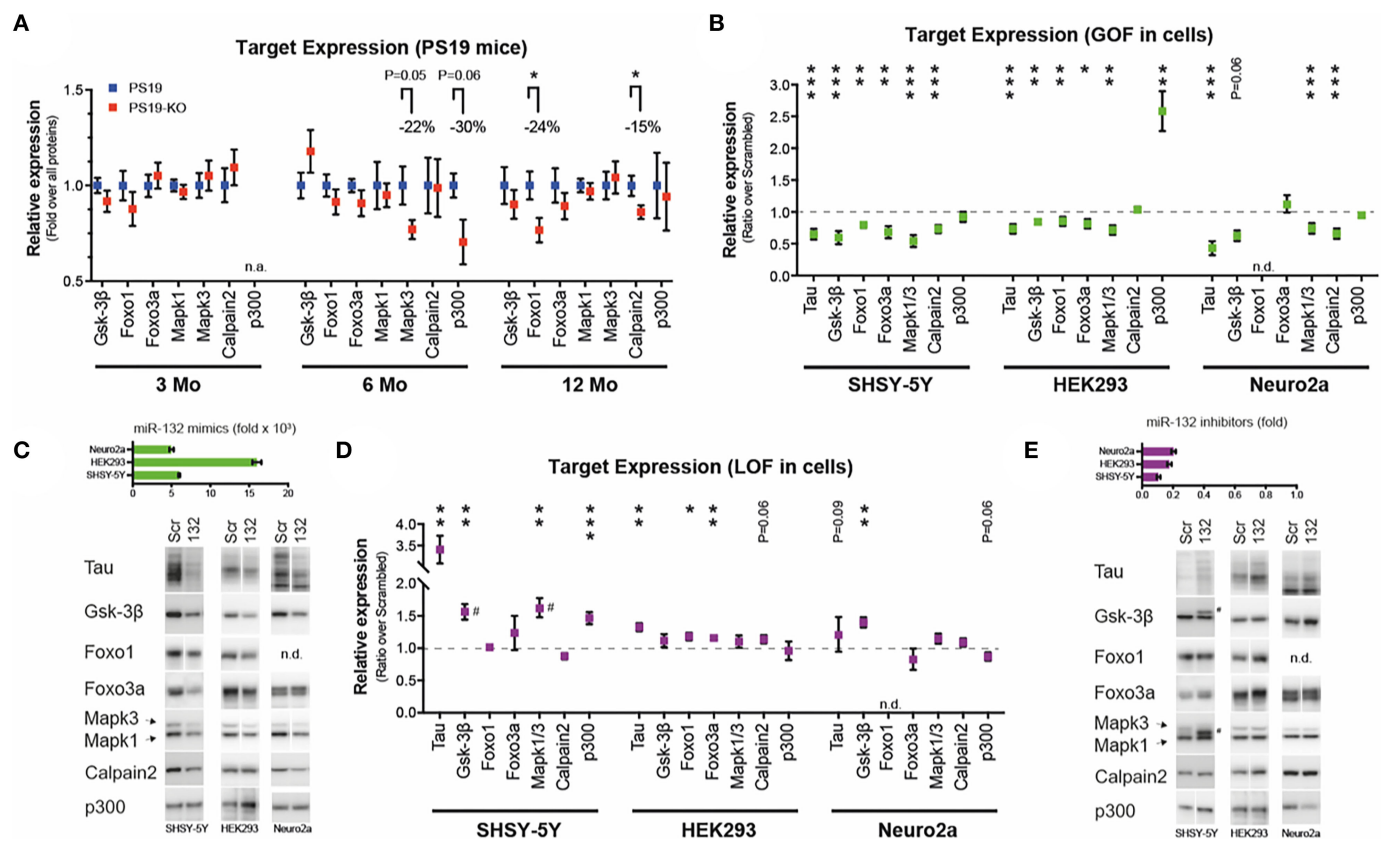
Analysis of miR-132 targets *in vitro* and *in vivo*. **(A)** Western blot quantifications of endogenous miR-132 targets (Gsk-3β, Foxo1, Foxo3a, Mapk1/Erk2, Mapk3/Erk1, Calpain2, p300) in PS19 and PS19-KO mice at different ages in the cortex. All significant trends and changes indicate lower expression in KO mice. *N* = 6–15 per group, mixed sex. Unpaired *t*-test, where *P < 0.05. **(B)** Western blot quantification of endogenous miR-132 targets, including tau, in native human SHSY-5Y, human HEK293, and murine Neuro2a cells. Cells were treated with 50 nM miR-132 mimics or scrambled control for 48 h prior to protein extraction. *N* = 2–4 experiments performed in triplicate. Results are shown as ratios between miR-132 over scrambled mimics (normalized to 1). Unpaired *t*-test, where *P < 0.05, **P < 0.01, ***P < 0.001. **(C)** Upper panel: Ectopic miR-132 levels measured by qRT-PCR in each cell line. Relative to scrambled mimic control. Lower panel. Representative western blot analyses. **(D)** Western blot quantifications of endogenous miR-132 targets. Here, cells were treated with 50 nM miR-132 inhibitors or scrambled control for 48 h. Unpaired *t*-test, where *P < 0.05, **P < 0.01, ***P < 0.001. **(E)** Upper panel: Endogenous miR-132 levels measured by qRT-PCR in each cell line following treatment. Relative to scrambled inhibitor control (normalized to 1). Lower panel. Representative western blot analyses. miRNA qRT-PCR were normalized using RNU19 as before (61, 64). Blots were normalized to all proteins using Ponceau or Stain-Free technology. # denotes possible alternate splicing (65) or phosphorylated form of targets. N.A., not available; N.D., not detected; GOF, gain-of-function; LOF, loss-of-function. Error bars represent SEM. In A, the groups of mice were analyzed separately per age. See Supplementary Methods for additional details.

We finally investigated a panel of miR-132/212 targets (other than tau) previously associated with tau metabolism or disease, including GSK-3β (26, 61), Mapk3/Erk1 (5, 64), Mapk1/Erk2 (5, 64), p300 (20, 26), Calpain 2 (26), Foxo1a (20), and Foxo3a (20, 26). We observed no significant differences caused by miR-132/212 deficiency on endogenous GSK-3β, Mapk1, and Foxo3a *in vivo* at all ages of study (Figure 2A). On the other hand, and unexpectedly, Mapk3, p300, Calpain2, and Foxo1a levels were reduced in PS19-KO mice, albeit at different ages (Figure 2A).

Of note, we could confirm the regulatory effects of ectopic miR-132 on all genes in cells (Figure 2B). Some cell-type specific effects were noticed, however, including an upregulation of p300 in miR-132-treated HEK293 cells. Representative miRNA qRT-PCR quantification and Western blots of targets are shown in Figure 2C. Inversely, inhibition of endogenous miR-132 in cells had only modest and sporadic effects on a subset of target genes (Figure 2D). Representative results are shown in Figure 2E. Clearly, some discrepancies exist in target gene regulation by miRNAs depending on models and methodological approach. Potential reasons for such paradoxes are discussed below.

### Barrier 1: Animal Models

To date, nearly 40 rodent models are available to study different tau species, isoforms and mutations (Alzforum.org) (66). In all cases, the study of human (or humanized) tau is essential to model human pathologies including aggregation and deposition. To our knowledge, only two rodent models contain the complete human tau 3′UTR. These include hTau mice (67) and MAPT knock-in mice (68). Obviously, only these or similar models can validate the direct binding of miRNAs to human tau mRNA. Only a few studies have investigated the effects of miRNA deficiency or overexpression on endogenous murine tau expression with its native 3′UTR (36, 61), but with no direct confirmation using miRNA seed mutagenesis or other techniques. Unfortunately, no group has yet evaluated the contribution of the human tau 3′UTR in disease progression and pathology in mice or other animals.

Of note, most findings linking miRNAs with tau were obtained in mice with human Aβ pathology (3xTg-AD, APP/PS1, 5xFAD) (36, 39, 61, 69). This cannot be underestimated since miR-132/212 deletion promoted tau aggregation in 3xTg-AD (61) but not (robustly) in PS19 mice (Figure 1G). Of course, we cannot exclude all other distinct features of each model (e.g., backgrounds, promoters, transgenes, disease onset) including model-specific variations in gene expression networks. The fact that PS19-KO mice display also lower tau phosphorylation and reduced (instead of increased) expression of some miR-132 targets could be accountable to these and other factors as well, including regulatory feedback loops and the absence of the human tau 3′UTR. Fortunately, recent advances in gene editing technologies *in vivo* (e.g., CRISPR-Cas9) and humanized models (e.g., induced pluripotent stem cells, knock-in mice) can help us to address these issues. The observation that other miRNAs can modulate tau pathology in PS19 mice (40) also opens the door to independent validation studies, that is, taking into consideration the pros and cons of transgenic mice without a human tau 3′UTR.

### Barrier 2: Physiological Regulation of tau and Other Targets

Proof-of-principle studies have shown that removing Dicer, the major enzyme responsible for miRNA maturation, induced significant changes in tau metabolism in both neurons (5, 19) and glia (70). However, this approach is largely inadequate to study single miRNAs and targets involved in tau regulation. The function of individual miRNAs is typically (and historically) inferred from overexpression studies in cells or animals, where a single miRNA can regulate tens (up to hundreds) of targets as predicted by bioinformatics tools. However, we know now that most overexpression studies do not reflect physiological context (47, 71–73). Indeed, they can result in the saturation of miRNA maturation products, induce off-target effects, or promote toxicity (74, 75). Lastly, and most importantly, gain-of-function (GOF) tells if a miRNA can exert a specific function, while loss-of-function (LOF) tests whether it is required for that function (72).

Interestingly, recent genetic inactivation studies in *C. elegans* suggest that some miRNAs function mainly through one or a limited number of master genes (76). Proof-of-principle exists in mice (for miR-155) but not yet in neuronal cells (77, 78). If true, most of the genes (modestly) influenced by miRNAs could be biologically “inactive” (47, 48). The results obtained herein *in vivo* (Figure 2A), often in theoretical disagreement with the mode of miRNA function, is somewhat in agreement with this hypothesis, more so given the subtle or negligible effects on tau aggregation and mouse phenotypes in PS19-KO mice. Ideally, the selection of dosage-sensitive genes, combined with prior documented effects on tau *in vivo*, would be important for functional validation studies.

A variety of technical and other biological factors need also to be considered in the future, including cell-type specificity, spatiotemporal regulation, statistical power, and potential compensation mechanisms from development or aging. Other tau modifications (e.g., acetylation) (79) could also influence its processing, expression, and analysis using more conventional antibodies. Adapted tools and models are now required to fully understand the regulation of tau by individual miRNAs in its physiological milieu, and at the single cell level.

### Barrier 3: Biological State and Context

There is increasing evidence that cell or biological context also influences miRNA function (80). The probability that miRNAs regulate tau differently according to the pathological state of the brain and peripheral system is therefore quite high. Contributing factors include inflammation, oxidative stress, immune response, and co-pathologies if present. Obviously, such systemic effects are difficult to reproduce in single animal models and even more in cells. And, of course, all of these factors influence miRNA expression to some degree. Interestingly, changes in the immune response and neuroinflammation are known to play a role on tau pathology in PS19 mice (62, 81). While some of these markers seem unaffected in PS19-KO mice (Figure 1H), a detailed analysis remains to be done. The challenge now is to decipher the individual and combined role of above-mentioned factors on miRNA-mediated tau pathology during disease onset.

### Barrier 4: Cause or Consequence

Understanding why, where, and when miRNAs become misregulated in tauopathies is key to elucidating their function and potential use in diagnostics and therapeutics. Interestingly, changes in miRNA levels occur at all stages of disease in tauopathy mouse models. Examples include miR-142, miR-10, miR-146, miR-155, miR-455, and miR-211 in THY-Tau22 mice (82), miR-142 in Tg4510 mice (83), miR-132, miR-146a, miR-22, and miR-455 in hTau mice (84), and miR-132 in PS19 mice (26). These results suggest that human tau (wildtype or mutant) itself can promote miRNA changes in the mammalian brain. The link with human disease remains, at this stage, uncertain given that most mouse models tested so far (THY-Tau22, Tg4510, PS19) overexpress human mutant tau.

The identification of causal mutations or risk factors within miRNA genes or binding sites provides an alternative strategy to elucidate the cause and consequence relationship between tau and miRNAs in humans. So far, there is little evidence that MAPT 3′UTR polymorphisms are associated with AD risk (85). Nevertheless, a role for 3′UTR polymorphisms in other tauopathies cannot be excluded. Interestingly, a study recently identified a polymorphism within the miR-142 promoter that confers risk for AD (86). Given the complexity of MAPT haplotypes and their importance in disease risk (87), one cannot exclude also a role for miRNA-mediated regulation in this context.

Another strategy to determine causality involves a cure or relief of disease symptoms using miRNA therapeutics. MiRNA mimics can rescue in part disease-related phenotypes in AD and tauopathy mice (40, 61, 69). As inferred above, it will be important to define a therapeutic window for the *in vivo* use of miRNA oligonucleotides in humans. Note that a miRNA replacement therapy, with the goal of restoring physiological miRNA levels, could provide an attractive alternative to overexpression *per se*. Taking into consideration all published studies so far, candidate miRNAs for therapeutics include, but are not limited to, miR-132, miR-142, miR-219, miR-455, and miR-146.

## Conclusion

To date, nearly 30 miRNAs have been implicated in the regulation of tau. The precise role and mode of action of individual miRNAs remains however unsettled. So far, the miR-132/212 cluster stands out for its potential role in regulating tau expression (11, 20, 26, 36, 61), splicing (19), acetylation (26), secretion (26, 88), proteolysis (26, 61), and aggregation (61). This adds to its correlation with disease progression and cognitive impairments in humans and mouse models. However, the choice of living model(s) and hypothesis(es) that need to be addressed are critical. Central elements of tau biology that have not yet been explored is the role of miRNAs in tau function (e.g., microtubule binding, DNA damage, gene expression, cell signaling) and propagation (e.g., spreading and seeding). The importance of miRNAs in familial tauopathies and many other sporadic tauopathies also remain unexplored. Clearly, much more work is needed to fully understand and appreciate the complexity of tau regulation by miRNAs and other non-coding RNAs.

## Data Availability Statement

The raw data supporting the conclusions of this article will be made available by the authors, without undue reservation. Requests should be directed to Sebastien.hebert@crchudequebec.ulaval.ca.

## Ethics Statement

The animal study was reviewed and approved by CHU de Québec Research Center Ethics Committee.

## Author Contributions

EB, JH-R, SR, SP, AL, CG, AT, IS-A, and RK planned and performed the experiments. EP provided valuable material and helped with experimental planning and writing. SH designed, planned and supervised the experiments, and wrote the article. All authors discussed the results and contributed to the final manuscript.

## Conflict of Interest

The authors declare that the research was conducted in the absence of any commercial or financial relationships that could be construed as a potential conflict of interest.
