# Supplementary material for: Advances and Challenges in Understanding MicroRNA Function in Tauopathies: A Case Study of miR-132/212
Source: Front Neurol. 2020 Sep 29;11:578720. doi: 10.3389/fneur.2020.578720 (PMC7553085; doi:10.3389/fneur.2020.578720)
Supplement: Supplementary file 1 [file Data_Sheet_1.PDF]

Supplementary Material

1 Supplementary Methods

1.1 Mice

PS19 transgenic mice overexpressing human mutant tau P301S (1N4R isoform) were purchased from the Jackson Laboratory (#008169). Generation of the full miR-132/212 knockout (KO) mice (a kind gift from Dr. R. H. Goodman, Vollum Institute, USA) was described previously (Magill et al., 2010; Hernandez-Rapp et al., 2015). Offspring from the 4th generation (F4) were used in all experiments, providing litter-matched PS19 heterogenous mice with or without the miR-132/212 cluster. Resulting control (PS19 +/-; miR-132/212 +/+, referred to as PS19) or miR-132/212-deficient mice (PS19 +/-; miR-132/212 -/-, referred to as PS19-KO) were scarified at either 3, 6 or 12 months. Note that the 12-month group also contained mice aged between 8 and 12 months that have died prematurely. The temperature was monitored just before sacrifice with a rectal probe (Thermalert TH-5; Physitemp, Clifton, NJ, USA). Mice were sacrificed by decapitation and the brains removed, dissected on ice and frozen on dry ice, as previously described (Hernandez-Rapp et al., 2016). All mouse studies were performed in accordance with the Université Laval ethics guidelines and regulations and approved by the VRRC Comité de protection des animaux committee.

1.2 Limb clasping

Mice were raised by the tail for 5 seconds over the home cage and scored for limb clasping on a scale of 0–5 (Levenson et al., 2016), where 0: No limb clasping; 1: one limb clasping; 2: two limbs clasping; 3: three limbs clasping; 4: four limbs clasping and 5: all limbs clasping severely.

1.3 Cell culture

Human neuroblastoma native cells (SH-SY5Y; ATCC #CRL-2266), Human embryonic kidney 293 (HEK293; from ATCC CRL-1573) and mouse neuroblastoma 2a (Neuro2a; from ATCC #CCL-131) and cultured in Dulbecco's modified Eagle's medium supplemented with 10% fetal bovine serum. Cells were incubated at 37°C with 5% CO2.

1.4 Cell transfection

Cells were seeded into six-well plates at 300,000 to 400,000 cells per well and incubated for 24 hours before transfection. For miR transfection, cells were transfected with 50nM of miR-132 mimics (#PM10166, Life Technologies, Burlington, Canada) or 50nM of scrambled miR mimics (Control) (#AM17110, Life technologies, Burlington, Canada). Conversely, we used either 50nM of miR-132 antagomiR (#MH10166, Life Technologies, Burlington, Canada) or 50nM of scrambled miR antagomiR (Control) (#4464076, Life technologies, Burlington, Canada). All transfections were performed using Opti-MEM I 1X (Gibco®, Life technologies, ref: 11058-021) and Lipofectamine 2000 (Life Technologies, ref: 11668-039, Burlington, Canada) as described by the manufacturers. Cells were harvested on ice and processed for experiments 48 hours post-transfection. Results are based on N=3-4 independent experiments in triplicate.

1.5 Protein and mRNA extraction

Total proteins were extracted as previously described (Boscher et al., 2019). Cells were rinsed with cold PBS then suspended in RIPA buffer (50 mM tris-HCL at pH 7.4, 150 nM NaCl, 1% NP-40, and 1mM EDTA) supplemented with phosphatase inhibitor (1mM activated sodium orthovanadate, 1mM sodium fluoride), 1mM phenylmethylsulfonyl fluoride, a complete mini EDTA-free protease inhibitor cocktail tablet (Roche life science) and 0.5% Sodium-deoxycholate (Sigma, cat n°D6750). Frozen mouse tissues were mechanically homogenized in 5x vol./weigh of RIPA buffer and lysed with a Sonic Dismembratod model 500 (Thermo Scientific). All lysates were incubated on ice for 20 min and centrifuged during 20 min at 20,000g at 4°C. The supernatant was removed and 15-20 µg of protein was mixed to the NuPAGE® LDS sample buffer (Life technologies) with a 5% final volume of β-mercaptoethanol for Western blot analysis. For the insoluble fraction, aliquots of supernatants were mixed to 1% N-Lauroylsarcosine sodium salt (Sarkosyl, Sigma) and incubated at 37°C for 1h on a rotarod. Then, the mixes were centrifuged 100000g at 20°C for 1h using Sorvall® mTX 150 Ultra Centrifuge (ThermoScientific). After centrifugation, the pellet was washed with 1% sarkosyl and dissolved in the NuPAGE® LDS sample buffer as previously described (Smith et al., 2015). Total RNA was extracted from cells using TRIzol reagent (Ambion by Life technologies, cat n°15596018) according to the manufacturer's instructions.

1.6 Western blotting

Fifteen-20 micrograms of protein were separated by TGX stain-free gel with Acrylamide 10% (Bio-Rad TGX stain-free FastCast Acrylamide kit 10%) and transferred onto a 0.45µm nitrocellulose membrane (Bio-Rad, Mississauga, Canada) as previously described (Boscher et al., 2019). The membrane was blocked with 5% non-fat milk and 1% BSA (Bovine Serum Albumin, Bioshop, ALB007-500) then incubated at 4°C overnight with the appropriate primary antibodies: Foxo1 (1:1000, C29H4, #2880, Cell Signaling), Foxo3a (1:1000, D19A7, #12829, Cell Signaling), Mapk1/3 (1:1000, L34F12, #4696, Cell Signaling), Calpain2 (1:1000, #2539, Cell Signaling), p300 (1:1000, D8Z4E, #86377, Cell Signaling), Tau CP27 (1:1000, gift of Peter Davies, Albert Einstein University, NY), PSD95 (1:1000, #2507, Cell signaling), NeuN (1:1000, MAB377, Millipore), Iba1 (1: 1000, #019-19741, Wako), GFAP (1:1000, SMI22, #835301, BioLegend), GSK-3β (1:1000, 3D10, #9832, Cell Signaling), Tau total (1:40 000, #A0024, Dako), Tau PHF1 (1:1000, gift of Peter Davies, Albert Einstein University, NY) and Tau pS422 (1:1000, ab9664, Millipore). On the second day, membranes were incubated with respective secondary anti-IgG-HRP antibodies (1:5000, anti-mouse code 115-035-146, anti-rabbit code 111-035-144, Jackson ImmunoResearch) at RT for 1h. The immune-reactive bands were acquired using Immobilon Western Chemiluminescent HRP Substrate (#WBKLS00500, Millipore) and visualized with the Fusion FX (Vilber Lourmat, Eberhardzell, Germany) imaging system. Normalization was done on total proteins obtained *via* Ponceau or TGX stain-free gel kit following manufacturer's instructions. Band intensities were quantified using the ImageJ software (Rueden et al., 2017).

1.7 miRNA qRT-PCR

MiR quantifications were done using the TaqMan miR Reverse Transcription Kit (Applied Biosystem, Burlingtom, Canada) and TaqMan Universal Master Mix (Applied Biosystem, cat n°4324018) following manufacturer's instructions. Primers were purchased from ThermoFisher (miR-132 ID: 000457; miR-99a ID: 000435). Mature miR-132 expression was normalized to miR-99a. The relative amounts of each transcript were calculated using the comparative Ct ($2^{-}\Delta\Delta$Ct)

method as before (Smith et al., 2011).

1.8 Statistical analysis

Unless otherwise stated, all statistical analyses were performed using GraphPad Prism 7 Software (Graph Pad Software, Inc, La Jolla, California, USA) as previously described (Boscher et al., 2019). Statistical differences were analyzed by the unpaired student's t-test, multiple t test, Kaplan-Meier or one-way ANOVA with multiple comparison (see figure legends) and p-values < 0.05 were considered to be statistically significant.

2 References

Boscher, E., Husson, T., Quenez, O., Laquerrière, A., Marguet, F., Cassinari, K., et al. (2019). Copy Number Variants in miR-138 as a Potential Risk Factor for Early-Onset Alzheimer's Disease. *J. Alzheimers Dis.* 68, 1243–1255. doi:10.3233/JAD-180940.

Hernandez-Rapp, J., Rainone, S., Goupil, C., Dorval, V., Smith, P. Y., Saint-Pierre, M., et al. (2016). microRNA-132/212 deficiency enhances Aβ production and senile plaque deposition in Alzheimer's disease triple transgenic mice. *Sci Rep* 6, 30953. doi:10.1038/srep30953.

Hernandez-Rapp, J., Smith, P. Y., Filali, M., Goupil, C., Planel, E., Magill, S. T., et al. (2015). Memory formation and retention are affected in adult miR-132/212 knockout mice. *Behavioural Brain Research* 287, 15–26. doi:10.1016/j.bbr.2015.03.032.

Levenson, J. M., Schroeter, S., Carroll, J. C., Cullen, V., Asp, E., Proschitsky, M., et al. (2016). NPT088 reduces both amyloid-β and tau pathologies in transgenic mice. *Alzheimer's & Dementia: Translational Research & Clinical Interventions* 2, 141–155. doi:10.1016/j.trci.2016.06.004.

Magill, S. T., Cambronne, X. A., Luikart, B. W., Lioy, D. T., Leighton, B. H., Westbrook, G. L., et al. (2010). microRNA-132 regulates dendritic growth and arborization of newborn neurons in the adult hippocampus. *Proceedings of the National Academy of Sciences* 107, 20382–20387. doi:10.1073/pnas.1015691107.

Rueden, C. T., Schindelin, J., Hiner, M. C., DeZonia, B. E., Walter, A. E., Arena, E. T., et al. (2017). ImageJ2: ImageJ for the next generation of scientific image data. *BMC Bioinformatics* 18. doi:10.1186/s12859-017-1934-z.

Smith, P. Y., Delay, C., Girard, J., Papon, M.-A., Planel, E., Sergeant, N., et al. (2011). MicroRNA-132 loss is associated with tau exon 10 inclusion in progressive supranuclear palsy. *Hum. Mol. Genet.* 20, 4016–4024. doi:10.1093/hmg/ddr330.

Smith, P. Y., Hernandez-Rapp, J., Jolivette, F., Lecours, C., Bisht, K., Goupil, C., et al. (2015). miR-132/212 deficiency impairs tau metabolism and promotes pathological aggregation *in vivo*. *Hum. Mol. Genet.* 24, 6721–6735. doi:10.1093/hmg/ddv377.
